# Analysis of the Human Breath

**Published:** 1881-11

**Authors:** 


### ANALYSIS OF THE HUMAN BREATH.

An account published in *Nature* of some experiments, made with a view to determine the organic matter of the human breath in health and disease, presents some facts of a peculiarly interesting nature. The breath of eleven healthy persons and of seventeen affected by disorders was examined, the persons being of different sexes and ages, and the time of day at which the breath was condensed varying. The vapor of the breath was condensed in a large glass flask surrounded by ice and salt at a temperature of several degrees below zero, the fluid thus collected being then analyzed for free ammonia, urea, and kindred substances, also for organic ammonia. Among the various results of this examination may be mentioned the fact that, in both health and disease, the free ammonia varied considerably; the variation, however, could not be connected with the time of day, the fasting, or the full condition. Urea was sought for in fifteen instances—three healthy persons and twelve cases of disease—but it was only found in two cases of kidney disease and in one case of diphtheria; and a faint indication of its presence occurred in a female suffering from catarrh. The quantity of ammonia arising from the destruction of organic matter also varied, possibly from the oxidation of albuminous particles by the process of respiration; but in healthy persons there was a remarkably uniformity in the total quantity of ammonia obtained by the process.

